# Successful Men

**Published:** 1874-11

**Authors:** 


### SUCCESSFUL MEN

Are those who make their own fortunes; this is the meaning of men of the world. Those who inherit fortunes usually die poor; hence that man makes the most permanent investment for his son who qualifies him for making his own fortune by providing him with an honorable calling, meanwhile instilling into his mind those principles of practice which are best calculated to win success. But as children are more deeply impressed with truths which they see in print than by the verbal communications of parents, a great point gained is to provide them with suitable reading in reference to the practical affairs of life in connection with impressive examples in the lives of men whose names are familiar to the public, and whose example is known to all and may be copied to advantage. Among self-made men are George Peabody, Stephen Girard, Cornelius Vanderbilt, Peter Cooper, Abraham Lincoln, President Grant, A. T. Stewart, and others. Sometimes the reading of a single sentiment in a newspaper makes an impression on the mind which tinges the whole subsequent life for good. Thirty years ago the elder Beach incorporated many short and valuable suggestions on conduct of life in his paper, the New York *Daily Sun*. The New York *Ledger* has done much good in this direction, and so does *Baldwin's Monthly* with its regular edition of fifty thousand copies.

A few years ago we brought the Boston *Journal of Chemistry* to the attention of our readers as a valuable family paper. It was then published at twenty-five or fifty cents a year, with only a few hundred subscribers; but such has been the ability of its editor, Dr. Nichols, that it goes to tens of thousands of families at a dollar a year. Among the items of a single old number are reliable statements on the use of glasses and spectacles, hints for preserving oil paintings, to transfer pictures to glass, softening old putty, how to know whether the colors of woven fabrics will stand, feeding animals, storing fruit, writers' cramp, functions of the liver, with a variety of suggestions for meeting the accidents and emergencies occurring in families, besides longer articles on interesting scientific subjects. Let parents and guardians see to it that those who are under their care, and for whose well-being they are responsible, are provided with solid, reliable, valuable, practical reading, such as is calculated to instruct them in the practical duties of life, and then they may expect them to grow up intelligent, useful, and influential members of society.

